# S10-3 The Global Observatory for Physical Activity-GoPA! Policy Inventory: Progress and methods

**DOI:** 10.1093/eurpub/ckac093.052

**Published:** 2022-08-29

**Authors:** Andrea Ramirez Varela

**Affiliations:** School of Medicine, Universidad de los Andes, Bogota, Colombia

**Keywords:** Physical activity, policy, national global

## Abstract

**Background:**

In 2015, GoPA! was launched to monitor progress on Physical Activity (PA) surveillance, policy and research globally. In 2017, the GoPA! started developing a PA Policy Inventory to enable collecting comparable data on PA policy worldwide.

**Methods:**

The instrument was developed in three stages. Stage-1: Critical assessment of the Policy Inventory version 1.0 was based on a review of other policy instruments/frameworks including: Health enhancing physical activity policy audit tool; the monitoring framework from the EU Recommendation on Health-Enhancing Physical Activity Across Sectors; and the Comprehensive Analysis of Policy on Physical Activity framework. Feedback from 14 GoPA! Country Contacts was received in 2017. Additional critical assessment was conducted from February to May 2019, and four PA policy experts drafted the Policy Inventory version 2.0. Stage-2: Open discussions about the Policy Inventory draft took place between May and August 2019 with seven policy experts. Based on the discussions, the draft questionnaire was revised three times to produce the GoPA! Policy Inventory version 2.0. Stage-3: Ten GoPA! Country Contacts provided their feedback on the Inventory version 2.0. The expert team from Stage 2 reviewed the suggestions and incorporated most of them into the GoPA! Policy Inventory version 3.0.

**Results:**

The GoPA! Policy Inventory version 3.0 contains: (i) a consent form; (ii) three questions about the respondent; and (iii) 20 questions about physical activity and sedentary behaviour policy. It is available in two formats: (i) an online survey in Qualtrics software; and (ii) an interactive Word document. The instrument collects the information related to: (i) national PA plans/policies; (iii) PA policy implementation; (iv) national recommendations on PA; (v) health surveillance or monitoring system that includes measures of PA; (vi) ministries or departments in national governments with an active role in PA promotion; (vii) quantifiable national targets related to PA; and (viii) comprehensiveness and effectiveness of the overall national PA policy.

**Conclusions:**

The instrument will be used to collect policy data for the 2020 GoPA! Country Cards to be launched at ISPAH2020 and, may further be used by PA researchers and policy analysts to monitor, audit, assess, and analyse national PA policies.

